# Electrical Injury and Wandering Atrial Pacemaker

**DOI:** 10.7759/cureus.18335

**Published:** 2021-09-27

**Authors:** Ranjan K Singh

**Affiliations:** 1 Internal Medicine, Anti-Retroviral Therapy Centre, District Hospital, Khagaria, IND

**Keywords:** wandering atrial pacemaker, voltage, electrical injury, arrhythmia, ampere

## Abstract

The supply of household electricity remains a low-voltage (110-220 V) energy source, and its effects on the human body depend on several factors, including the type of contact and duration of contact, among other things. In a significant number of cases, direct contact with household electricity causes reversible cardiac arrhythmia-ventricular fibrillation, ventricular premature beats, atrial tachycardia, and atrial fibrillation.

Wandering atrial pacemaker (WAP) is a benign atrial arrhythmia observed in elderly patients suffering from obstructive pulmonary diseases that result from an ischemic heart. This report discusses WAP as observed in a patient who suffered an electrical injury.

## Introduction

The effects of electrical injury vary from skin burn to internal organ damage directed especially at the cardiovascular and nervous systems. The extent of electrical injury depends on the type of electricity source, i.e., direct current (DC) or alternating current (AC), the duration of contact with the source of electricity, the state of the body whether wet or dry, the presence of calluses over the palm, the route of electrical flow, and the level of voltage [1]. The severity of an electric shock depends on the current flow (I) measured in ampere (A). It is linked to the resistance of the conductor (R, unit: ohm ‘W’) and the potential difference between the two ends of a conductor (Volt; unit V), and is derived by applying the formula based on Ohm’s law: i.e., I = V/R. The severity of an electrical burn, by contrast, depends on the energy (Watt) and is derived from Joule’s formula W=I2 x R x T (duration of exposure with the source of current).

Household electrical supply is a low-voltage (220 V) AC at 60 Hz frequency. The physiological effects of contact with a low-frequency AC (60 Hz) current vary at different amperes. For example, 1mA (1/1000 A) is barely perceptible as numbness, whereas 20 mA can cause respiratory muscle paralysis, while 100 mA reaches a threshold for ventricular fibrillation [1,2]. The resulting cardiac arrhythmia may take the form of ventricular fibrillation, ventricular tachycardia, ventricular premature beats, atrial premature beats, atrial arrhythmia, and/or heart block [2].

## Case presentation

A 40-year-old male patient was brought into the emergency ward after suffering an accidental electrical injury that involved an entry wound in the middle of his left hand and an exit wound in the back of his chest. He was holding the hanging rod for a ceiling fan when the connection was plugged in, resulting in electric shock. He lost consciousness and fell to the ground with the rod clenched in his hand for a minute and a half. The electricity source was disconnected and cardiopulmonary resuscitation was administered to the patient by his neighbors. The patient regained consciousness and complained of aching all over the body along with general weakness. 

**Figure 1:**
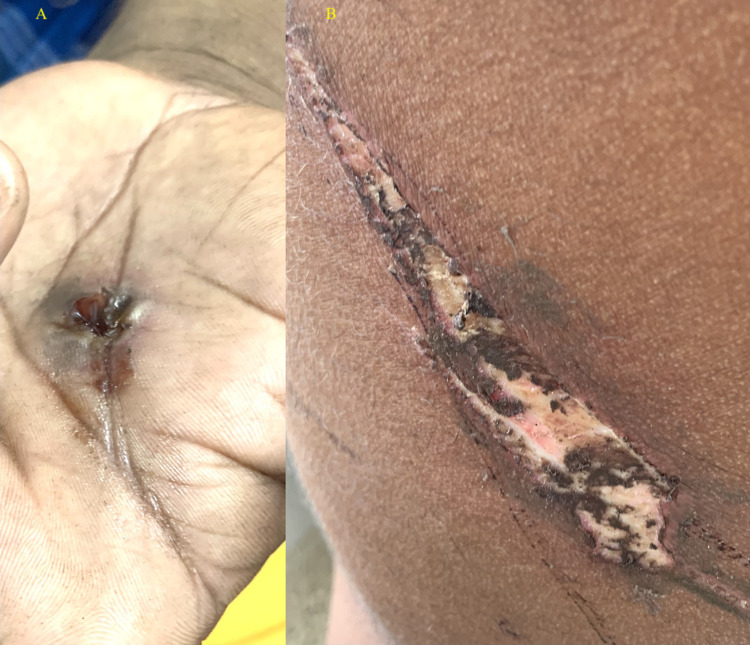
Entry wound (panel A) and exit wound (panel B).

He had a black hole in the middle of his left palm (Figure 1A) and a linear burn on the back of his chest (Figure 1B).

**Figure 2:**
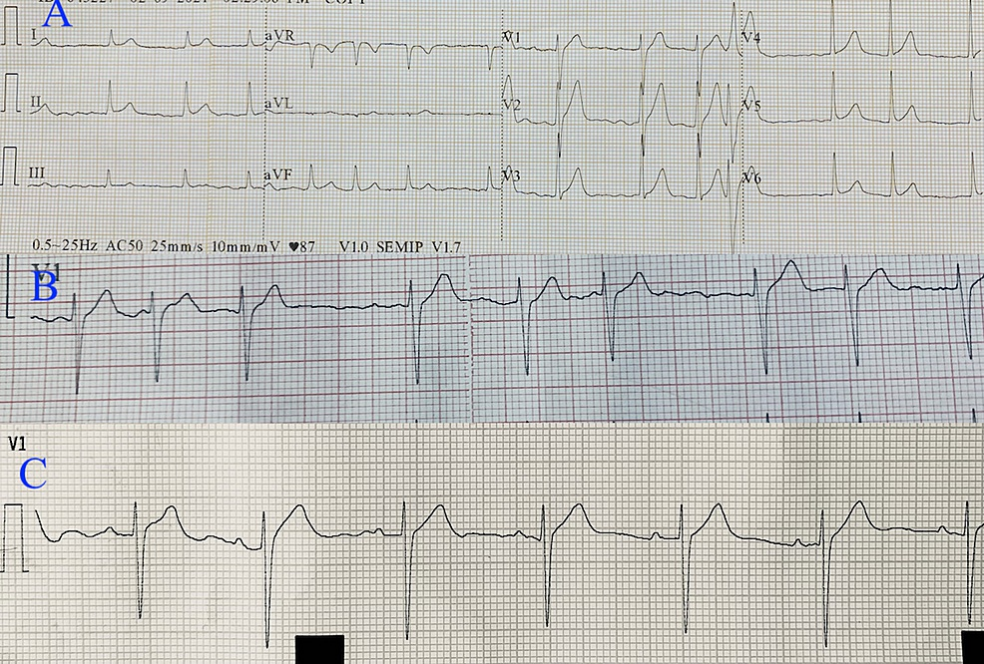
Panel A shows the ventricular premature beat in V1-3, panel B shows wandering atrial pacemaker, and panel C shows sinus rhythm.

His pulse was irregularly irregular at 78/minute, and his blood pressure was 110/78 mm Hg. His total leucocyte count was 8600/cmm with neutrophils at 64%, and his hemoglobin was 13 gm/dL. Urinalysis did not show myoglobin. Serum sodium and potassium were 134 mEq/L and 4.2 mEq/L, respectively. Electrocardiography (ECG) showed occasional ventricular premature beat with wandering atrial pacemaker (Figures 2A-2B). Of note, the patient did not have any kind of cardiac ailment previously. The patient was hydrated with intravenous fluids and his wounds were treated with antiseptic dressings and antibiotics. He remained under observation for 48 hours and the ECG showed sinus rhythm (Figure 2C).

## Discussion

Low voltage currents cause severe electrical burns to the skin as a result of high energy output from the current flow. Dry skin with callouses over palm (resistance of 500 W) and a long contact of palm with the source of electricity attribute to severe burn in this patient (Joule formula). Thereby, the electrical energy output is dissipated and there is less internal injury [3,4].

Low voltage currents travel through the body along low-resistance pathway nerves and blood vessels to cause severe cardiac injury. Also, the distance between the entry and exit wounds can determine the severity of the cardiac injury. The heart remains in the central location of the electrical current’s pathway between the left palm and back of the chest. Current spikes occur in the palm and fingers of an individual holding a metal rod that is suddenly connected to an electric source [5]. The electric shock causes depolarisation of cardiac muscles and increases membrane pores of the cells resulting in arrhythmias; sinus tachycardia, ventricular premature beats, ventricular tachycardia, and atrial fibrillation are common [6,7]. Wandering atrial pacemaker (WAP) is a benign atrial arrhythmia that has been observed in this case study. WAP and multifocal atrial tachycardia (MAT) differ only with the heart rate - WAP has a heart rate less than 100 bpm whereas MAT has a heart rate greater than 100 bpm. In the WAP rhythm, the pacemaker wanders with the impulses originating from the sinoatrial node to the atrium, and to the atrioventricular junction with a changing focus. Hence, the P waves on an ECG are presented in different configurations. WAP is differentiated from sinus arrhythmia by the fact that heart rate variability occurs from beat-to-beat, and is not phasic. Also, in sinus arrhythmia, the P-wave morphology and the P-R interval are constant [7]. Most of the arrhythmias occur soon after electric shock and are short-lived. However, delayed arrhythmias occurring 12 hours after electric shock have been reported, too [8].

## Conclusions

Household electric supply is low voltage AC of 60 Hz. It is the electric current that determines the pathophysiological effects in the body but the voltage does determine the outcome of electric shock. Even a low-voltage shock can cause ventricular fibrillation if resistance is low and current flow reaches a threshold of 100 mA. The severity of burn lesion is determined by the resistance of skin and duration of exposure with the source of current. Most cardiac arrhythmias are short-lived and do not require treatment.
